# Recent Progress of the Practical Applications of the Platinum Nanoparticle-Based Electrochemistry Biosensors

**DOI:** 10.3389/fchem.2021.677876

**Published:** 2021-05-03

**Authors:** Han Yu, Jingbo Yu, Linlin Li, Yujia Zhang, Shuquan Xin, Xiuzhen Ni, Yuan Sun, Kai Song

**Affiliations:** ^1^School of Life Sciences, Changchun Normal University, Changchun, China; ^2^Center of Pharmaceutical Engineering and Technology, Harbin University of Commerce, Harbin, China

**Keywords:** biomolecules, biosensors, noble metals, platinum nanoparticles, applications

## Abstract

The detection of biomolecules using various biosensors with excellent sensitivity, selectivity, stability, and reproducibility, is of great significance in the analytical and biomedical fields toward achieving their practical applications. Noble metal nanoparticles are favorable candidates due to their unique optical, surface electrical effect, and catalytic properties. Among these noble metal nanoparticles, platinum nanoparticles (Pt NPs) have been widely employed for the detection of bioactive substances such as glucose, glutamic acid, and hormones. However, there is still a long way to go before the potential challenges in the practical applications of biomolecules are fully overcome. Bearing this in mind, combined with our research experience, we summarized the recent progress of the Pt NP-based biosensors and highlighted the current problems that exist in their practical applications. The current review would provide fundamental guidance for future applications using the Pt NP-based biosensors in food, agricultural, and medical fields.

## Introduction

**Scheme 1:**
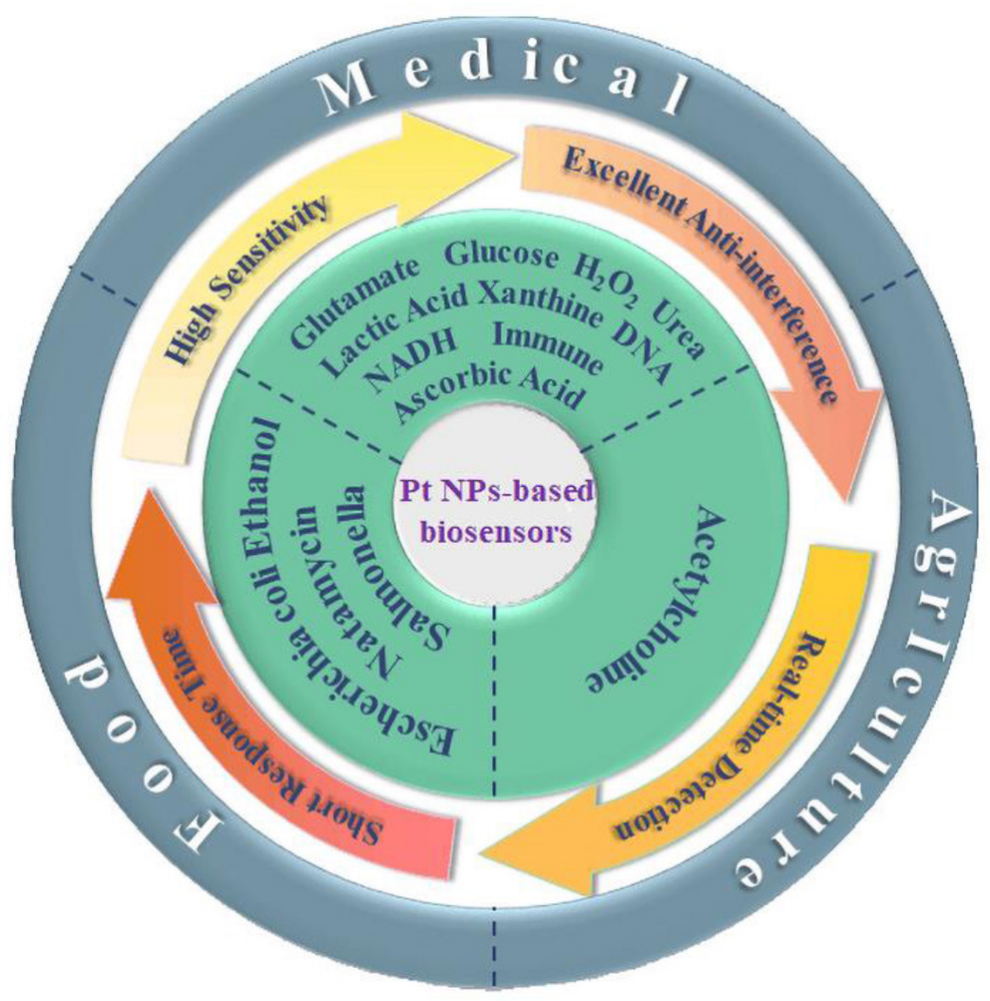
The applications of Pt and the Pt-based nanomaterials in electrochemical sensors in food, agricultural, and medical fields.

Since their advent in 1977, biosensors have undergone significant technological advancements typically from surface measurement to subcutaneous implantation, carrier-mediated enzyme sensors to non-carrier bioelectrocatalytic sensors, as well as organic metals to noble metal nanomaterials. Electrochemical biosensors are one of the most representative of all biosensors. The detection principle is based on antigens/antibodies, enzymes, nucleic acids, aptamers, and other biometric identification elements, to capture the target; thus, causing changes in the current, impedance, potential, or conductance of the sensor surface. The most common detection methods are cyclic voltammetry (CV), differential pulsed voltammetry (DPV), alternating current voltammetry (ACV), square wave voltammetry (SWV), electrochemical impedance spectroscopy (EIS), electrochemiluminescence (ECL), and photoelectrochemical chemistry (PEC). Performance evaluation of sensors, such as detection limit, sensitivity, response time, linear range, stability, selectivity, and reproducibility (Table 1), mainly depends on the performance of the sensing element. Noble metal nanomaterials have been used as sensing elements of electrochemical biosensors because of their good biocompatibility, excellent electrical and thermal conductivity, excellent chemical stability, and large specific surface area. Platinum nanoparticles have unique properties such as their surface effect, volume effect, quantum size effect, and macroscopic quantum tunneling effect, and can be synthesized by various methods to obtain materials with different physical and chemical properties (Nowak, 2013; Borman, 2016; Choi et al., 2017; Wang et al., 2018). The chemical synthesis method accurately controls the size and shape of nanoparticles and affords strong surface chemical versatility and high yield. In contrast, physical methods achieve specific nanoparticle properties based on the control of pulses, temperature, and ambient gas pressure. The bio-assisted synthesis method can be prepared on a large scale without toxic reaction solvents and avoids a complex laboratory setup. Platinum nanomaterials on biosensors are mainly used in three ways: as electrochemical catalysts to accelerate the reaction of enzymes, as sensor electrodes to enhance electron transfer, and as to modify the electrode surface into an enzyme to fix the substrate and maintain enzyme activity (Zhai et al., 2013; Krishnan et al., 2017; Uzunoglu and Ipekci, 2019). Biosensors based on the Pt nanomaterials, such as amperometric acetylcholinesterase biosensors for pesticide detection, electrochemical biosensors using platinum nanotubes for glucose detection, as well as graphene platinum-based hydrogen peroxide biosensors for reactive oxygen species (ROS) detection, have been widely used in the past few decades (Scheme 1) (Zhang et al., 2014; Yang et al., 2017; Ma et al., 2019). In view of this, the current review summarized the development and application of the Pt nanoparticle-based biosensors and further highlighted their potential applications in the food, agricultural, and medical sectors.

**Table 1:** Evaluation indexes of sensor performance.

**Evaluation indexes**	**Evaluation function**
Detection limit	The highest technical performance index specified by an instrument or method to test an object (i.e., gas, liquid, solid). It is the limit that can be achieved by the corresponding instrument or method
Sensitivity	Refers to the degree of change in response to a method to changes in the unit concentration or unit quantity of the substance to be measured. It can be described by the ratio of the response of the instrument or other indicators to the concentration or quantity of the corresponding substance to be measured
Response time	Refers to the time required for the change of the measured object to reach the sensor output
Linear range	Refers to the interval between the maximum amount and the minimum amount of the variation range of the concentration of the test substance which is linear and has a method to obtain the test results with precision and accuracy to meet the requirements
Stability	The ability of a sensor to maintain its metering characteristics constantly over time
Selectivity	Only the target detection is carried out and is not easily disturbed by impurities
Reproducibility	Refers to the degree to which the measurement results of the same measured quantity are consistent when they are tested under different measurement conditions

## The Applications of the Pt NP-Based Biosensors in Agricultural Fields

With the development of nanotechnology based on platinum nanoparticles, composite nanometer material is used in the preparation of electrochemical biosensors, and the detection of pesticide residues particularly phosphate ester compounds in organophosphorus pesticides, with low detection limit, high analyte selectivity, and high universality.

### Detection of Acetylcholine

Organophosphorus pesticides (OPs) are broadly used in agricultural pest control methods (Dong et al., 2016; Lu et al., 2018); however, the use of pesticide residues often causes severe environmental pollution (Peng et al., 2017; Jiang et al., 2018). Environmental monitoring techniques such as gas chromatography (GC), liquid chromatography (LC), mass spectrometry (MS), and capillary electrophoresis (CE) are common methods to detect environmental pollution (Bucur et al., 2018; Uniyal and Sharma, 2018); however, alternative tools such as acetylcholinesterase (AChE) amperometric biosensors are sensitive, accessible, and cost-effective (Song et al., 2017; Zhou et al., 2017), compared with the previous methods.

**Figure 1:**
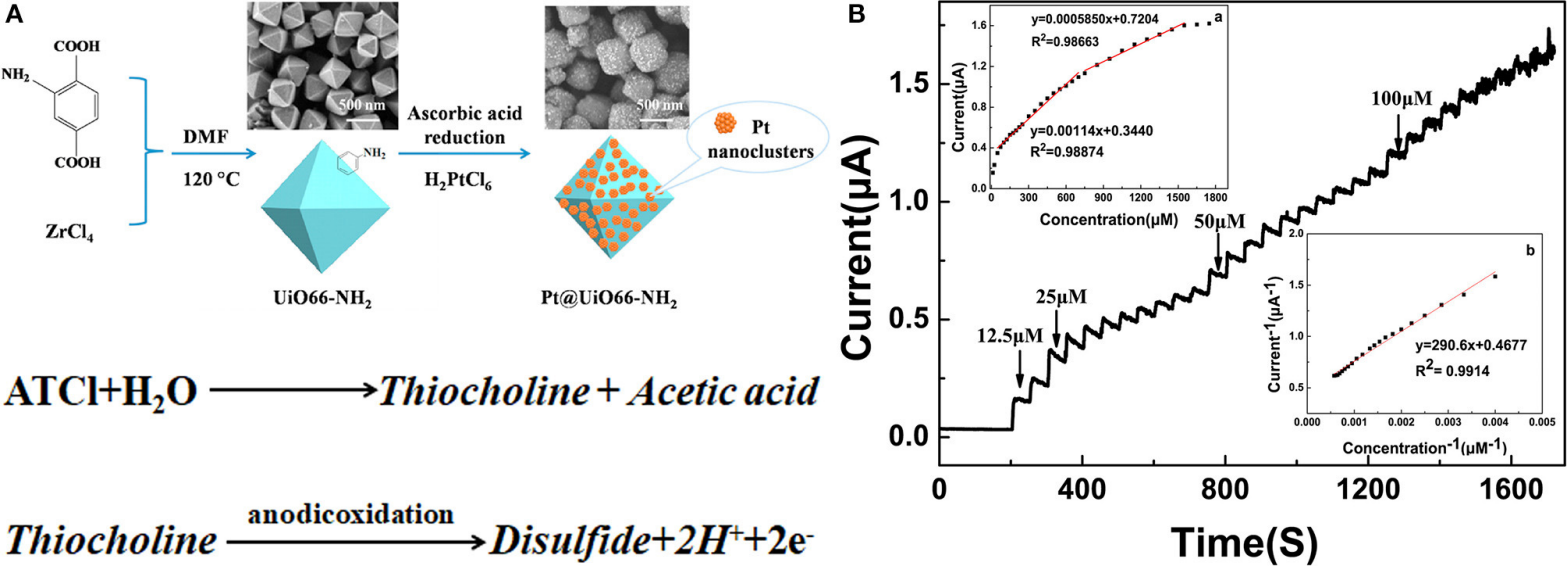
**(A)** The synthesized process of Pt@UiO66–NH2. **(B)** The current response of the AChE/Pt@UiO66–NH2/GCE when ATCl is continuously after the addition to PBS (0.1 M, pH 7.5) at 650 mV (Sun and Wang, 2010). Reproduced with the permission from 2019 Elsevier Ltd.

Li et al. (2016) first coated a glassy carbon electrode with a nanocomposite film following amplification and the bromophenol blue doped molecular imprinted polymer (IMP), prepared the electrochemical insecticide imidacloprid (IMI) sensor, and was applied to the detection of vegetables. However, the performance indexes of the sensor are not given. Ma et al. (2019) synthesized Pt@UiO66–NH_2_ by mixing zirconium-based organic skeleton nanomaterials (UiO66–NH_2_) and chloroplatinic acid (H_2_PtCl_6_), with ascorbic acid and deionized water. The resultant Pt@UiO66–NH_2_ had a large specific surface area and high dispersibility, and therefore can be used as AChE biosensors (Figure 1A). In the absence of organophosphorus pesticide, the substrate and AChE can catalyze the conversion of acetylthiocholine chloride (AtCl) into an electroactive substance thiocholine, which then can be converted into electronic signals. In contrast, the presence of OPs phosphorylates AChE can form a stable complex that can inhibit AChE activity, leading to AChE conversion and AtCl reduction (Peng et al., 2017; Lu et al., 2018). The longer the binding time of OPs is, the more stable the complex, with the optimum time being 300 s (Figure 1B) (Sun and Wang, 2010). Represented by the organophosphorus insecticide marathon, the differential pulse voltammetry response (DPV) has an oxidation peak at 0.65 V, and the peak current decreased with the increase in concentration. The detection range of the sensor was 1 × 10^−14^–1 × 10^−9^ M, while the detection limit was 4.9 × 10^−15^ M. The sensor can directly detect organophosphorus pesticides in the environment and food such as cabbage and apple samples.

However, some problems remain to be solved for agricultural biosensors based on platinum nanomaterials. First, the development process is complex. The preparation of platinum-based biosensors involves toxic substances and high concentrations of enzymes, which increases the production cost of the sensing platform. Second, these biosensors have disadvantages of a single pesticide detection quantity, unstable fixed device material, and overall stability and reproducibility that leave much to be improved. At the same time, there is a gap among many designs and practical applications; therefore, the development of a simple, stable, cheap, non-toxic immobilized substrate, especially for on-site detection of organophosphorus pesticides, deserves great attention.

## The Applications of the Pt NP-Based Biosensors in Medical Fields

Pt NP-based biosensors play an important role in the field of medicine (Table 2). Traditional test methods have complex operations and long cycles, which cannot meet the needs of clinical medicine. Biosensors make it possible to establish rapid testing. Platinum-based biosensors can be used to efficiently and rapidly detect various chemical components in body fluids, such as lactic acid, uric acid, and glutamic acid, along with many carcinogens.

**Table 2:** Summary of applications of platinum nanoparticles in electrochemical biosensors.

**Sample**	**Detection limit**	**Linear range**	**Sensitivity**	**Response time**	**References**
Glutamate	0.1 μM	0.004–0.9 mM	973 ± 4 μA mM^−1^cm^−2^	—	Barman et al., 2017
	0.03 μM	1–925 μM	5.73 ± 0.078 nAμM^−1^ mM^−2^	<1 s	Nguyen et al., 2020
Glucose	—	—	103 μA mM^−1^cm^−2^	—	Wang et al., 2005
	2.5 mM	—	—	5 s	Zhu et al., 2006
	6.7 M	0.1–1 mM	2.527 A/mM	—	Wu et al., 2007
	0.055 mM	0.16–11.5 mM	—	—	Wen et al., 2009
	—	0.1–8 mM	0.94 μA mM^−1^ cm^−2^	—	Feng et al., 2014
	1.21 μM	2–10 mM	27.51 μA mM^−1^cm^−2^	—	Akkaya et al., 2018
	0.2 μM	0.025–2.2 mM	45.2 μA mM^−1^cm^−2^	—	Yang et al., 2017
	1.8 μM	0.006 mM	64.51 μA mM^−1^cm^−2^	<40 s	Uzak et al., 2020
	Alkaline solution: 3.2 μM Neutral medium: 28 μM	7.2 mM	Alkaline solution: 24.6 μA mM^−1^ cm^−2^ Neutral medium: 2.1 μAmM^−1^ cm^−2^	—	Liu et al., 2012
	0.01 μM	12 μM	3,577 μA mM^−1^cm^−2^	—	Dhara et al., 2014
	0.052 μM	0.025–12 mM	40.9 mA mM^−1^cm^−2^	2 s	Savk et al., 2020
H_2_O_2_	—	0.01–2 mM	33.66 μA mM^−1^cm^−2^	—	Sanzo et al., 2017
	0.86 ± 0.19 μM	0.8 μM−8.6 mM	1,381 ± 72 μA mM^−1^cm^−2^	—	Wang et al., 2019
	~0.2 M	0.5–3.475 mM	45 9 ± 3 mAM^−1^cm^−2^	<5 s	Zhang et al., 2014
	0.1 μM	1–900 μM	—	—	Chen et al., 2018
	0.5 μM	1–1,477 μM	341.14 μA mM^−1^cm^−2^	—	Liu et al., 2015
Lactic acid	6.9 μM	—	41,302 ± 546 μAM^−1^ cm^−2^	—	Loaiza et al., 2015
	—	1–20 mM	1.43 μA mM^−1^	50 s	Liu et al., 2009
NADH	4.78 and 6.18 μM	25 and 21 mM	68.24 and 40.21 μA mM ^−1^cm^−2^	—	Jimenez et al., 2014
Xanthine	PV FXO-: 1.73 × 10^−3^–1.74 mM; PV FXO-/PT: 0.43 × 10^−3^ −2.84 mM	PV FXO-/PT: 0.43 × 10^−3^–2.84 mM	—	—	Bas et al., 2011
Ascorbic acid	1.9 × 10^−5^ M	—	—	—	Dursun and Gelmez, 2010
	0.796 μM	1 μM−0.6 mM	—	—	Wang et al., 2017
Urea	—	0.7–26.7 mM	0.153 mA mM^−1^cm^−2^	—	Hosseinian et al., 2019a
	2.57 mM	PPy electrode: 1.67–8.32 mM; MPPy electrode: 0.5–10.82 mM	PPy electrode: 0.0035 mA mM^−1^; MPPy electrode: 0.0432 mA mM^−1^	PPy electrode: 7 s; MPPy electrode: 5 s	Hosseinian et al., 2019b
DNA	1 × 10^−9^ M	14 × 10^−9^-2.14 × 10^−7^ M	—	—	Wang et al., 2008
	—	—	2.4 nM	—	Yin et al., 2012
	0.6 fM	1.0 fM−10 pM	—	—	Dong et al., 2012
	4.3 × 10^−5^ M	1 × 10^−6^–100 × 10^−6^ M	—	—	Singhal et al., 2017
	2 × 10^−15^ M	1 × 10^−14^–5 × 10^−9^ M	—	—	Daneshpour et al., 2016
Immune	—	0.01–1 μg ml^−1^	184.8 Ω cm^2^	—	Mishra et al., 2014
	CEA: 0.021 ng/ml; RAC: 0.051 ng/ml; Thrombin: 2.4 ng/ml; Hg^2+^: 0.22 ng/ml	CEA: 0.025–1.6 ng/ml; RAC: 0.0625–4 ng/ml; thrombin: 4–128 ng/ml; Hg^2+^: 0.25–16 ng/ml	—	—	Fu et al., 2017
	1–7 log CFU/ml	—	—	—	Malvano et al., 2018
	91 CFUml^−1^	4.0 × 10^−2^–4 × 10^−8^ CF∙ml^−1^	—	—	Zhu et al., 2018a
	10 ng/ml	—	—	—	Long et al., 2020

### Detection of Glutamate

L-glutamate acid is one of the main components of mammalian neurotransmitters, and its concentration imbalances can cause psychiatric disorders such as Alzheimer's disease and Parkinson's disease (Barman et al., 2018). The detection of glutamate levels in the extracellular space of brain tissues is therefore essential in the evaluation of psychiatric disorders. The conventional methods for the detection of glutamic acids, such as chromatography, fluorescence, spectrophotometry, and electrochemiluminescence, are not only time-consuming and laborious, but also require specialized skills (Sanchez and Gallardo, 1992; Chovin et al., 2004; Acebal et al., 2008; Shah et al., 2008; Knittl et al., 2015). The use of the electrochemical method for glutamate detection is simple, low-cost, and gives real-time results (Barman et al., 2017).

Following Hummer's method (Hummers and Offeman, 1958), Barman et al. (2017) developed an electrochemical L-glutamic acid biosensor from graphene oxide (Crbxl-RGO) and Pt nanoparticles. They first developed Crbxl-RGO and immobilized the composite material in the sensing region. The stability of the immobilized glutamate oxidase was then enhanced by 1-ethyl-3-(3-dimethylaminopropyl)-carbodiimide [EDC] treatment and –COOH group activation (Bartczak and Kanaras, 2011). The sensor has a sensitivity of 973 ± 4 μA/mMcm^2^, a linear range of 0.004–0.9 mM, a detection limit of 0.1 μm, and showed excellent specificity and selectivity.

**Figure 2:**
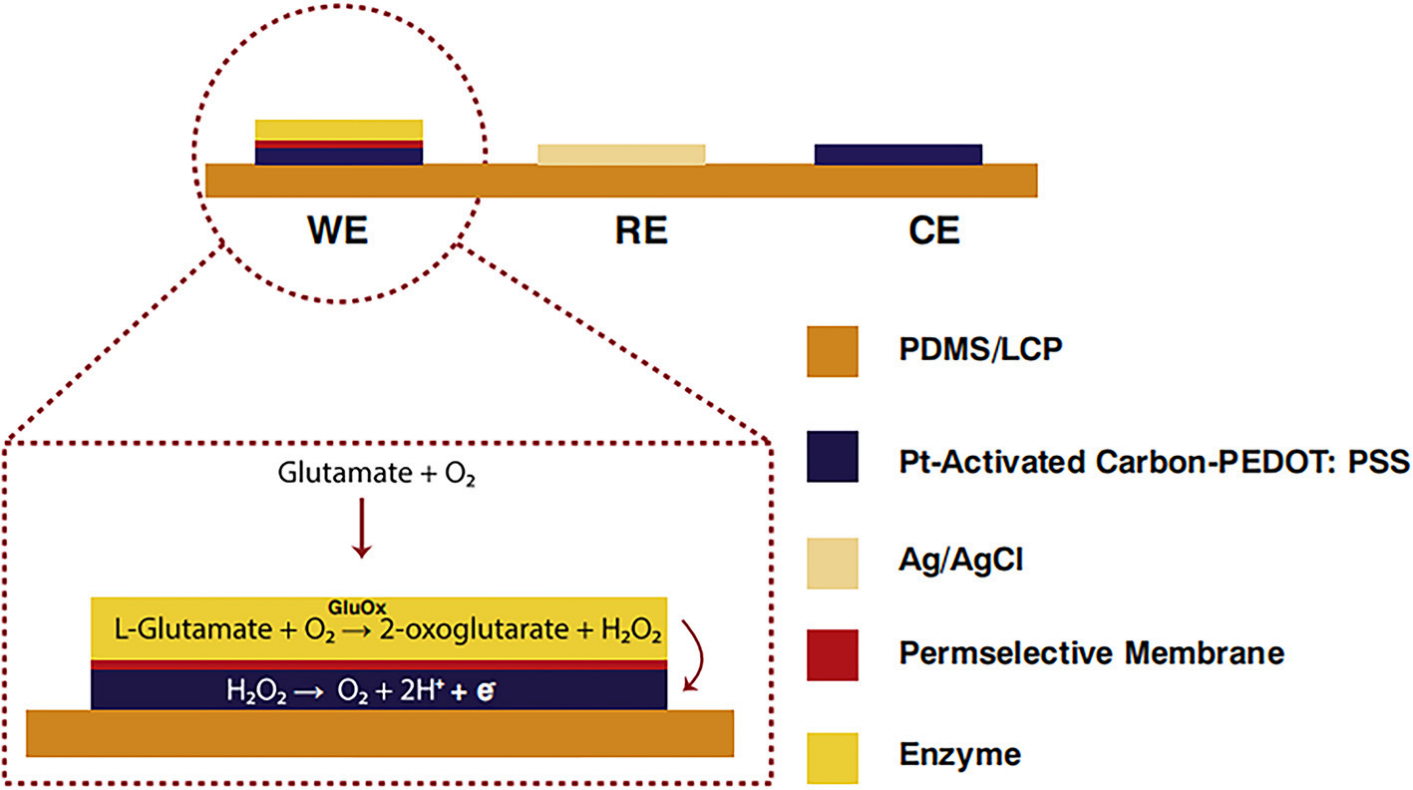
Glutamate biosensors: A cross-sectional view of C-PT-PEDOT [poly (3,4 ethylenedioxythiophene)] (Nguyen et al., 2020). Reproduced with the permission from 2020 Elsevier B.V.

Nguyen et al. (2020) generated current on the surface of an electrode by converting glutamic acid into H_2_O_2_ using commercial activated carbon and platinum particles and catalyzed by glutamate oxidase (Figure 2). This resulted in glutamate with a short response time, high sensitivity, and low detection limit. It was equipped with astrocytes that are highly sensitive to glutamate. Particularly, it has a sensitivity of 5.73 ± 0.078 nAμM^−1^mm^−2^, a detection limit of 0.03 μm, a response time of <1 s, and a linear range of 1–925 μm.

Moreover, the sensitivity of the sensor can be greatly improved and can be used for *in vivo* detection. Metal oxide-based glutamate sensors have even detected glutamate successfully without the presence of enzymes; however, enzyme-free glutamate sensors for platinum nanomaterials *in vivo* and in the food industry are still under development. The development of new enzyme-free sensors for platinum-based nanomaterials with good selectivity, high sensitivity, portability, *in vivo* detection, and commercial application, is therefore the key direction of future research and development.

### Detection of Glucose

The diagnosis and prognosis of diabetes depend largely on glucose levels in the blood (Khanh et al., 2014). Besides that, glucose concentration is also essential in food, beverage, and fermentation industries (Sungur et al., 2014). Glucose oxidase (GOx) played an important role in the development of glucose biosensors in the study of Clark and Lyons (1962).

In 2004, an electrooxidated amperometric biosensor based on H_2_O_2_ was developed. The biosensor was produced by oxidases and enzyme-catalyzed oxidation of the analyte (Wang et al., 2005). Inclusion of GOx in polypyrrole (PPY) membrane on a platinum electrode provides an effective way for enzyme immobilization and a barrier for ascorbic acid and dopamine. In fact, the sensitivity of the sensor is 103 μAmM^−1^cm^−2^.

Despite the high sensitivity of the sensor, its response time and detection limit still remain non-negligible. To further clarify the performance of the glucose sensors, Zhu et al. (2006) exploited the thermal, electronic, and photonic properties of carbon nanotubes (CNTs) to coat Pt (diameter of 2–3 nm), and formed dendrimer- encapsulated Pt nanoparticles (Pt-DENs) coupled with multiwalled carbon nanotubes (MWCNTs). The MWCNTs do not only serve as a solid phase for adsorption and concentration of metal nanoparticles, but also promote electron transfer reactions with enzymes and other biomolecules (Guiseppi-Elie et al., 2002). The electrode assumed high redox activity and minimizes interference. These data showed a response time and detection limits of 5 s and 2.5 mM, respectively, whereas while the development of the biosensor is simple and easy to control, its detection limit is not satisfactory.

**Figure 3:**
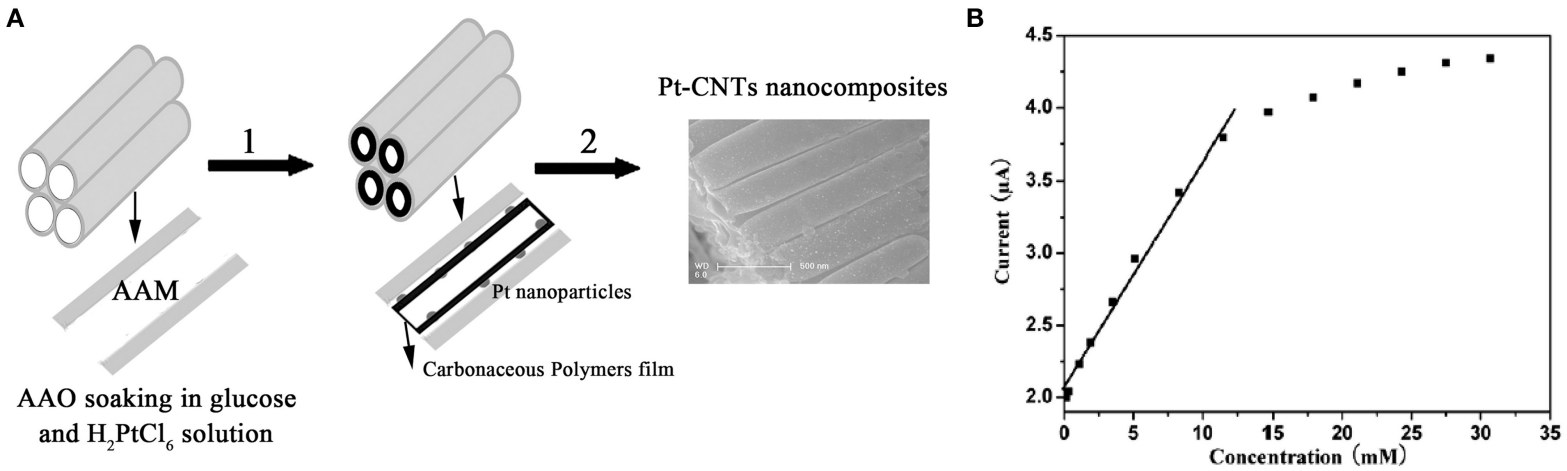
**(A)** Schematic diagram showing the steps for the synthesis of the Pt-CNTs. **(B)** The calibration curve of steady-state current vs. glucose concentration (Wen et al., 2009). Reproduced with the permission from the American Chemical Society.

Meanwhile, Wu et al. (2007) prepared a glucose biosensor using MWCNTs, gold nanoparticles, and glucose oxidase (GOD). The sensor had a detection range of 0.1–10 mM, a sensitivity of 2.527 A/mM, and detection of 6.7 M. Wen et al. (2009) devised a simple method for the preparation of the Pt-CNT. They carbonized glucose and a reductant on anodic aluminum oxide film nanocomposites to maintain the biological activity of GOD (Figure 3A). The composite contained many oxygen-rich groups, which can improve water solubility and biocompatibility, and maintain the biological activity of the GOD (Yang et al., 2006). The mixing of the composite material with the GOD allowed the generation of a high-performance glucose electrochemical biosensor, based on the Pt-CNT. The biosensor had a linear range of 0.16–11.5 mM with a detection limit of 0.055 mM, as well as decent consistency and specificity (Figure 3B).

Feng et al. (2014) used a simple physical adsorption method to prepare glucose sensors using graphene-modified TiO_2_ nanotube arrays (TiO_2_ NTAS), platinum nanoparticles (Pt/GR/TiO_2_ NTAs), and immobilized glucose oxidase (GOx). Its response rate linearly correlated with glucose concentration from 0.1–8 mM. Its sensitivity reached 0.94 μA·mM^−1^·cm^−2^, up from XYZ.

**Figure 4:**
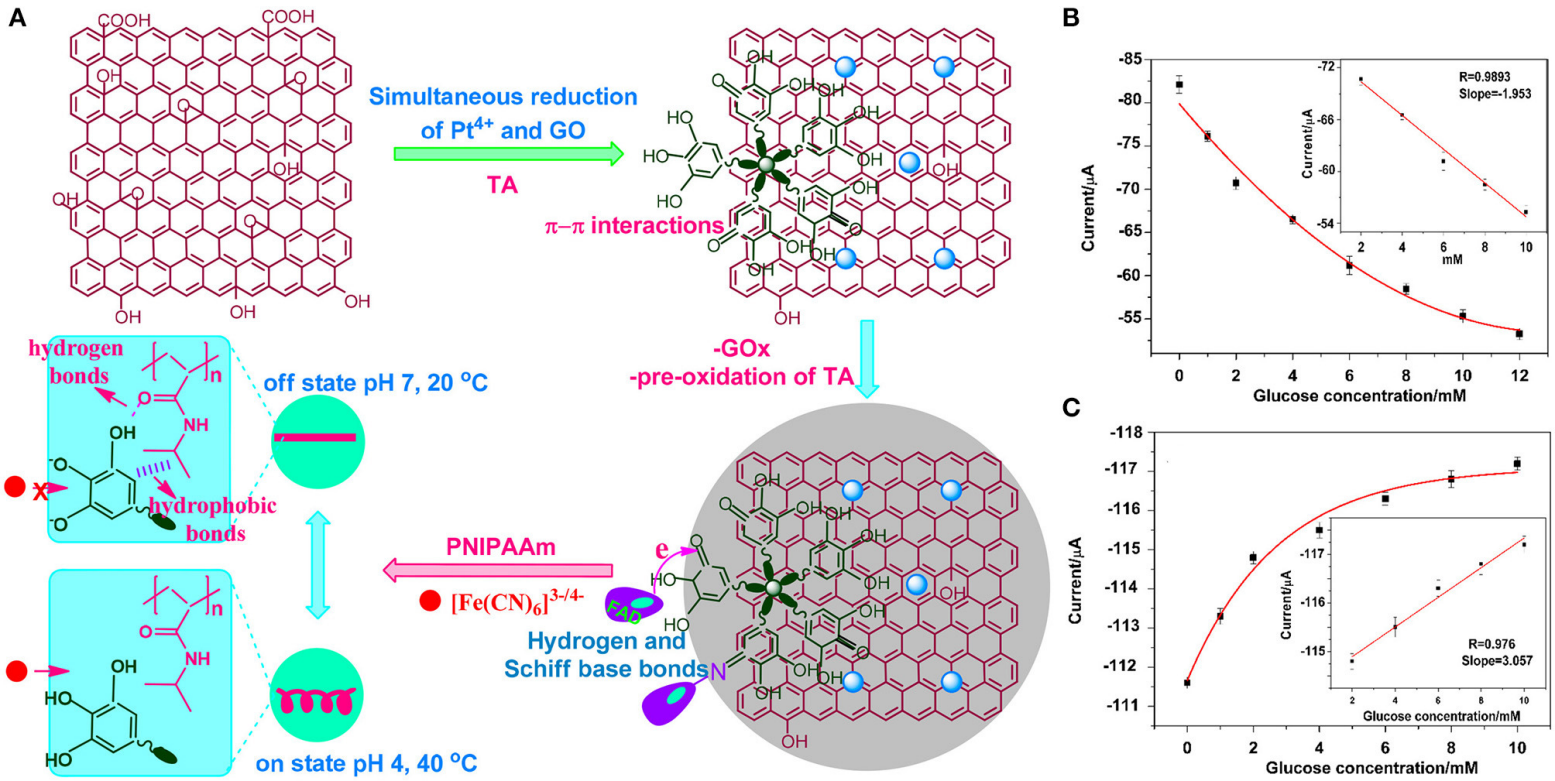
**(A)** Fabrication of an electrochemical switch biosensor. **(B)** The change of cathode current of the GO increases glucose concentration within particular concentration range. **(C)** Switch biosensor sensitivity, detection limit, and linear measurement (Akkaya et al., 2018). Reproduced with the permission from the American Chemical Society.

Akkaya et al. (2018) invented a novel switching electrochemical biosensor which was prepared based on the composite modification of Pt nanoparticle deposition and reduction graphene oxide. They also applied tannic acid (TA) to simultaneously reduce Pt^4+^ and graphene oxide (GO), in order to immobilize Gox. This resulted in a switchable surface that can respond to changes in pH, oxygen, and temperature. In addition, the biosensor has a polyisopropylacrylamide (PNIPAAm) which can be connected via hydrogen bonds to form a zipper-like switchable biosensor (Figure 4A). At a concentration between 2–10 mM, the GOx flavin adenine dinucleotide (FAD) current decreased with the increased glucose concentration (Figure 4B); thus, the reversible interaction confirmed that the biosensor has good repeatability, detection limit (1.21 μM), and sensitivity (27.51 μAmM^−1^cm^−2^) (Figure 4C).

To further reduce the detection limit and improve sensitivity, Yang et al. (2017) immobilized the nanocoated material in GOx solution and prepared a series of one-dimensional (1D) mesoporous platinum nanotubes (MPtNTs) as electrode modifiers to 1D mesoporous Pt nanotubes modified biosensor (MPNB). The MPtNTs had wall thicknesses ranging from 7.2–12.8 nm. Mesoporous nanoparticles are suitable for enzyme immobilization, with high specific surface area and capacity for enzyme activity (Zhao et al., 2013; Zhu et al., 2015). Here, the response current to glucose increased with concentration. The MPtNTs biosensor, with a wall thickness of 7.2 nm, had a sensitivity of 45.2 μA·mM^−1^·cm^−2^ (3.19 μA·mM^−1^), a detection limit of 0.2 μM (S/N = 3), and a linear range of 0.025–2.20 mM. In addition, the estimated Michaelis constant (Km) is <3.4 mM, which confers good anti-interference and selectivity.

With the development of biosensors and the optimization of various indicators such as response time, sensitivity, or detection limit, crystalline porous material with a periodic network structure composed of organic linkers, metal ions, and metal-organic frameworks (MOFs) have been developed (Strauss et al., 2019). They have adjustable pore sizes, ultra-high porosity, and large hydrophilic/hydrophobic groups (Majewski et al., 2017; Hu et al., 2018; Xie et al., 2018). In fact, the MOF-74 group is considered a relatively strong framework material with decent thermal and water stability (Burtch et al., 2014; Luo et al., 2019).

**Figure 5:**
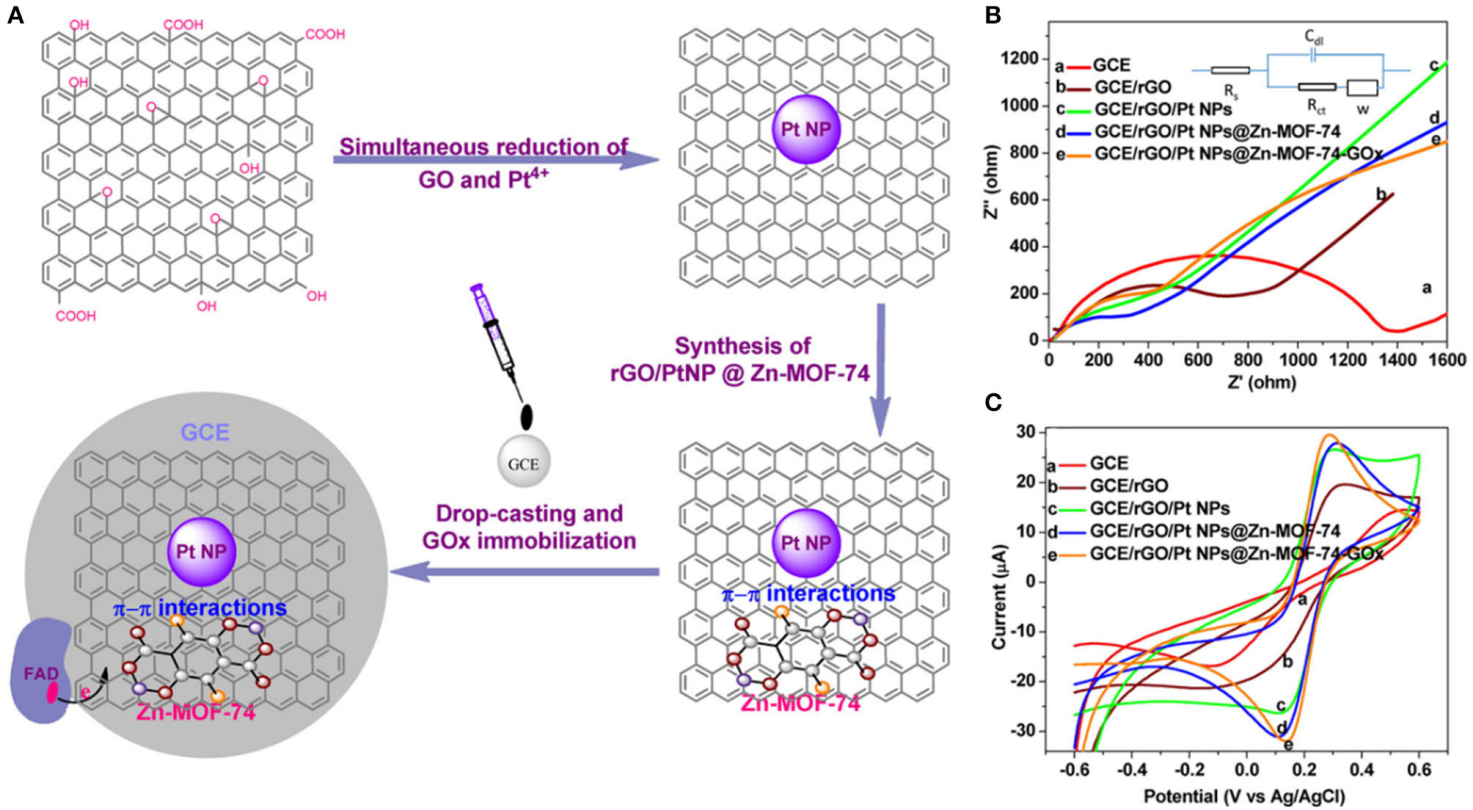
**(A)** Development of reduced GO/Pt nanoparticles/Zn-MOF-74 nanomaterials. **(B)** Nyquist plot of the electrodes and the Randles circuit as well as cyclic voltammograms of the electrodes in 0.1 M PBS containing 5 mM [Fe(CN)6]3-/4- and **(C)** 0.1 M KCl, with a scan rate of 100 mVs^−1^ (Uzak et al., 2020). Reproduced with the permission from 2019 Wiley.

On the other hand, Uzak et al. (2020) immobilized the GOx on platinum PtNP-modified reduced graphene oxide (RGO)/Zn-MOF-74 hybrid nanomaterial (Figure 5A). The biosensor generates high current density, is rapid, and sensitive to glucose, with efficient electron transfer capacity (Figures 5B,C). The data showed that the stable current signal can be obtained within 40 s. In the presence of various interference reagents, the relative standard deviation (RSD) of the current change reaches 4.16% with good selectivity. The linear range of the biosensor is 0.006 mM, while the detection limit and sensitivity are 1.8 μM (S/N = 3) and 64.51 μAmM^−1^cm^−2^, respectively.

Enzyme-based sensors are relatively sensitive to operating conditions (pH, humidity, and temperature), costly, and unstable (Savk et al., 2020). These limitations have led to the development of enzyme-free glucose sensors. To further optimize the performance of the glucose biosensors, Liu et al. (2012) used platinum nano corals and platinum nanofibers, and modified electrodes using gold particles to prepare sensors for directly detecting glucose in neutral and alkaline solutions. The sensor was highly sensitive to the alkaline solution (24.6 μA mM^−1^ cm^−2^), exhibited a low detection limit (3.2 μM), and displayed a wide linear range of 7.2 mM. In a neutral medium, both the linear range and sensitivity (2.1 μAmM^−1^ cm^−2^), as well as detection limit, increased to 28 μM.

Dhara et al. (2014) prepared an RGO serum glucose sensor using cubic crystal platinum nanomaterials and monoclinic crystal copper oxide nanomaterials based on a one-step chemical method. The detection limit, sensitivity, and linear response of the sensor were 0.01 μM, 3,577 μAmM ^−1^cm^−2^, and 12 μM, respectively.

Savk et al. (2020) synthesized activated carbon-loaded Pt–Ni nanocomposites (PtNi/AC). The biosensors were modified on the surface of the glassy carbon electrode (GCE) by PtNi/AC to enable the development of an enzyme-free electrochemical glucose biosensor. PtNi/AC can effectively catalyze the oxidation of glucose in the absence of an enzyme. The addition of glucose significantly changes the intensity of the current. The current density increased steadily, reaching a stable current in 2 s. The sensitivity reaches 40.9 mAmM^−1^cm^−2^, the linear range 0.025–12 mM, and detection limit 0.052 μM.

Platinum-based glucose biosensors should be developed from the following aspects. First, traditional glucose meters are suitable for detecting human blood but require invasive methods to collect samples. The resulting discomfort of test takers necessitates the development of a noninvasive sensing platform. Second, nanotechnologies that provide enhanced electroactive surface area and superior sensitivity, such as metals or metal oxide nanostructures, have accelerated the development of platinum-based enzyme glucose sensors with excellent analytical performance. They are, however, limited in terms of biocompatibility, lifetime, and selectivity. Future development of glucose biosensors should be aimed at developing platinum-based regenerative biosensors, or should be developed in combination with other materials (e.g., molecularly imprinted polymers, ligand, peptide arrays, and adhesions). This direction suggests that a more standardized assessment of the analytical performance of glucose biosensors needed to achieve accurate and reliable testing.

### Detection of H_2_O_2_

Reactive oxygen species (ROS) are important intracellular signaling molecules that can regulate protein synthesis and cell apoptosis (Chang et al., 2013; Zhang et al., 2013). However, the excessive accumulation of the ROS can lead to oxidative stress and further causes autoimmune diseases, Alzheimer's disease, and cancer (Trachootham et al., 2009; Wu et al., 2011; Pagliari et al., 2012). Hydrogen peroxide (H_2_O_2_) is a typical ROS in the cellular environment. Through the selective quantitative analysis of H_2_O_2_ and the evaluation of its dynamic release process, we can fully understand its key role in cell physiology (Chang et al., 2013; Zhang et al., 2013).

Sanzo et al. (2017) used Pt (II) and platinum nanospheres and Pt (IV), and platinum nanoflowers to prepare modified gold nano corals and platinum hybrid bimetallic nanostructures, respectively, with different shapes and sizes. The nano coral gold was first coated with Pt nanospheres and platinum nanoflowers and thereafter cross-linked with glucose oxidase and glutaraldehyde hydride on the bimetallic Au–Pt nanostructure. The composite effectively detected hydrogen peroxide. Its detection range was between 0.1–2 mM whereas the sensitivity was 33.66 μA/mM cm^−2^; however, the sensitivity of the composite for hydrogen peroxide needs further improvement. Wang et al. (2019) prepared an oxidase-based hydrogen peroxide biosensor through electrodeposition of Pt nanoparticles on carbon fiber microelectrodes. The Pt–carbon hybrid sensor had a sensitivity of 7,711 ± 587 uA·mM^−1^cm^−2^, a detection limit of 0.53 ± 0.16 μM, and a linear range of 0.8 μM−8.6 mM. The sensitivity and detection limit of the hybrid sensor against hydrogen peroxide were 1,381 ± 72 μA·mM^−1^·cm^−2^ and 0.86 ± 0.19 μM, respectively.

Even though enzyme-based hydrogen peroxide sensors have long been used, enzyme-free sensors are emerging alternatives.

**Figure 6:**
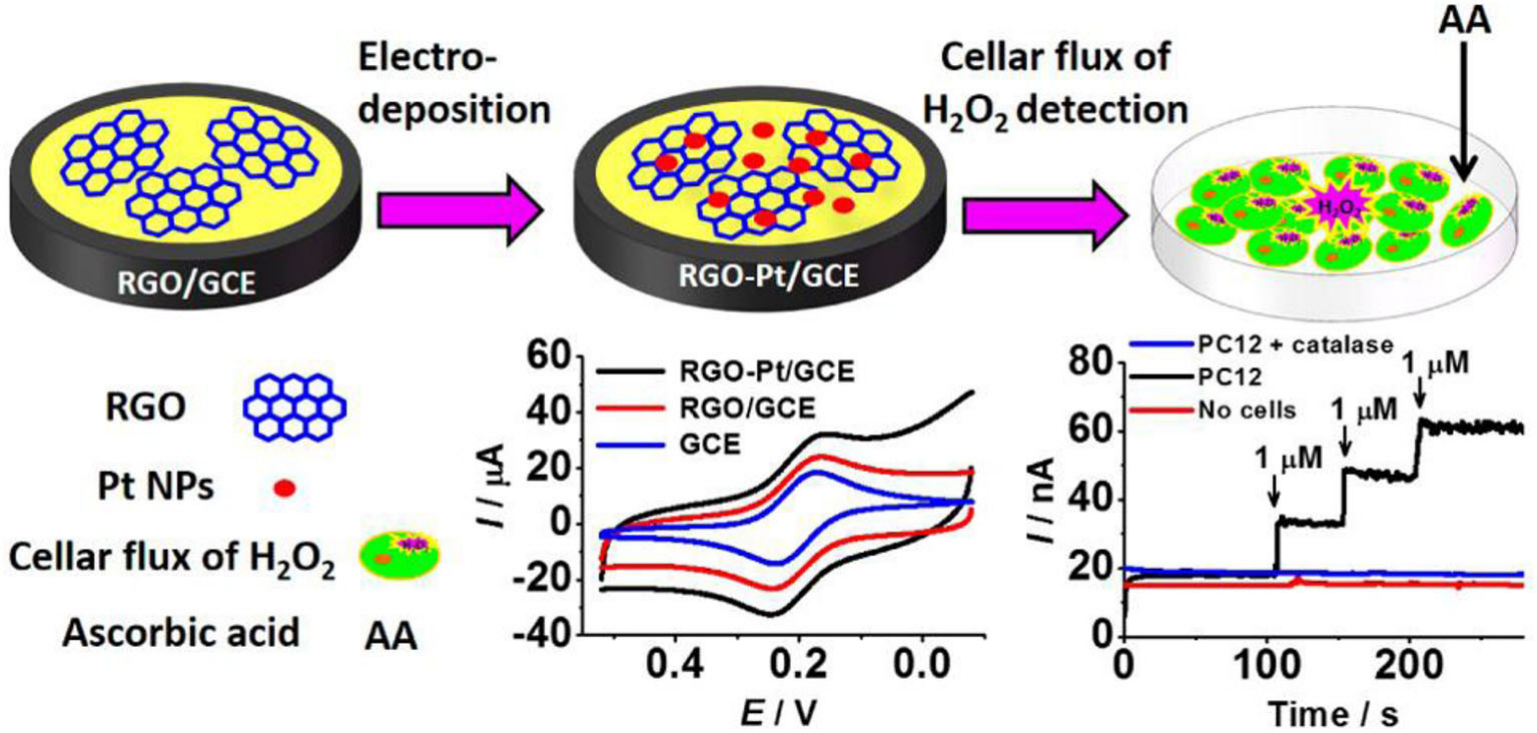
rGO–Pt-modified GCE was used to detect H_2_O_2_ outflow from cells stimulated by ascorbic acid (AA) (Zhang et al., 2014). Reproduced with the permission of the American Chemical Society.

Zhang et al. (2014) constructed a sensor which could measure H_2_O_2_ concentration in living cells using graphene–platinum (rGO–Pt) nanocomposites (Figure 6). Within 5 s following the injection of H_2_O_2_, the steady-state current of RGO-Pt/GCE reached 95%. The linear response of H_2_O_2_ ranges from 0.5 to 3.475 mM, while the detection limit is ~0.2 M, and the sensitivity is 459 ± 3 mAM^−1^ cm^−2^; thus, the sensor is highly resistant to interference.

Based on the rGO–Pt nanocomposites, Zhao et al. (2017) fabricated an amperometric H_2_O_2_ biosensor using iron oxide-reduced graphene oxide (Fe_3_O_4_/rGO) nanocomposite (Hummers and Offeman, 1958; Wu et al., 2012; Sanzo et al., 2017; Zhao et al., 2017; Wang et al., 2019). They electrodeposited the Pt with a GCE. These data showed that the biosensor had fast amperometric response to H_2_O_2_, with a linear range of 0.1–2.4 mM (*R*^2^ = 0.998), a sensitivity of 6.875 μA/mM, and a detection limit of 1.58 μM (S/N = 3). After 30 days, the current response maintained at 96.8% of the original one, showing long-term stability and high selectivity.

**Figure 7:**
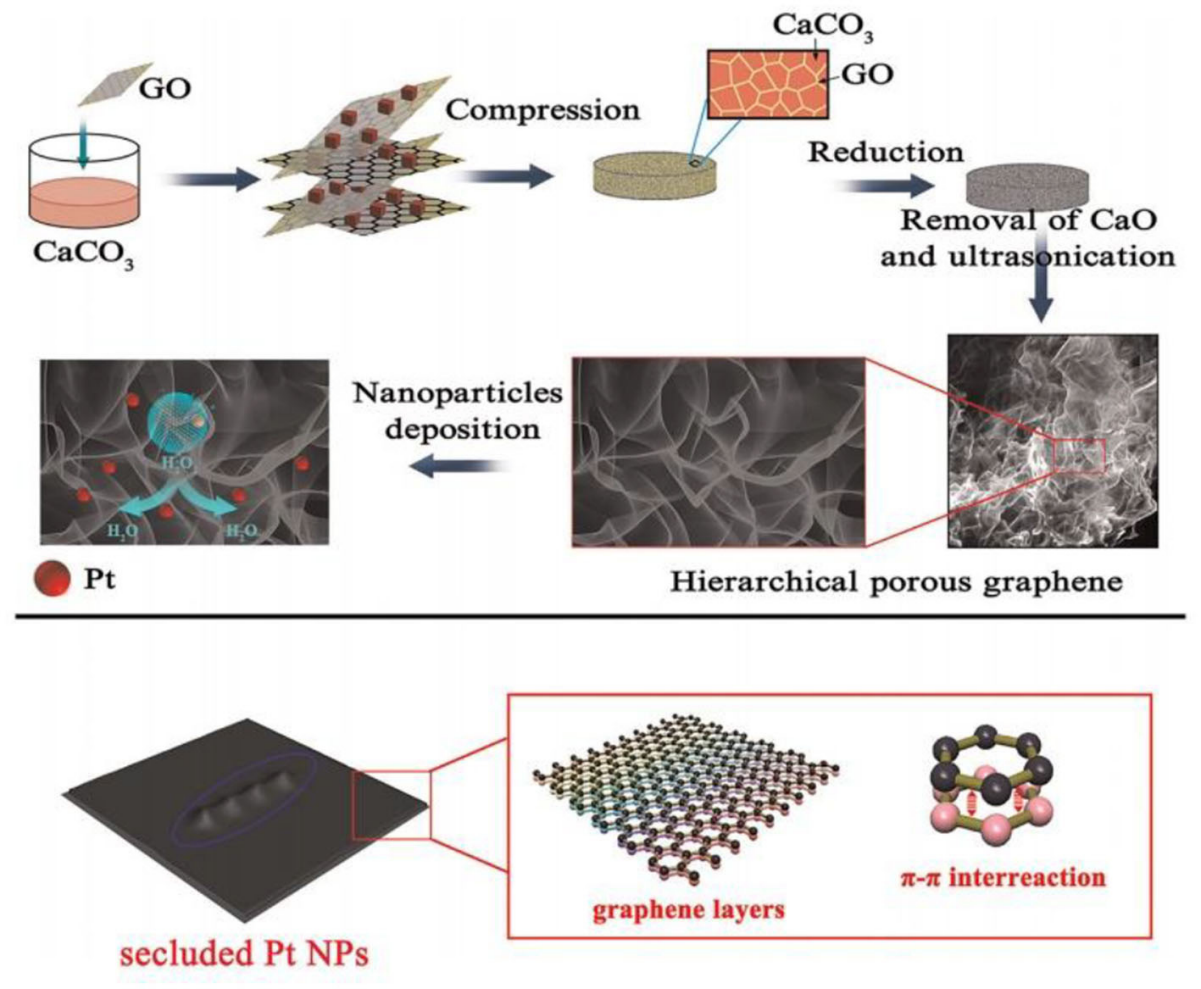
Schematic flow for the preparation of Pt/porous graphene samples (Liu et al., 2015). Reproduced with the permission of 2015 Elsevier B.V.

Using Pt nanoparticles/carbon quantum dots/ionic liquid functionalized graphene oxide (PTNPS-CdS/IL-GO) nanocomposites, Chen et al. (2018) prepared a hydrogen peroxide sensor with a linear range of 1–900 μM and a detection limit of 0.1 μM. To lower the detection limit, Liu et al. (2015) used a microwave-assisted method to coat porous graphene with platinum nanoparticles (Figure 7). The modified sensor had a detection linear range of 1–1,477 μM, a sensitivity of 341.14 μA mM^−1^ cm^−2^, and a detection limit of 0.5 μM. It also exhibited good anti-interference performance, consistency, and prolonged stability.

Pt nanoparticles prepared by the electrodeposition method can significantly reduce the overpotential of H_2_O_2_ and enhance the electron transfer rate between the electrode and the Pt nanoparticles. Compared with the horseradish peroxidase electrochemical biosensor, the biosensor prepared by electrodeposition of Pt and different nanomaterials has the advantages of strong anti-interference ability, good stability, and strong applicability, which enables more convenient and accurate determination of H_2_O_2_ content.

### Detection of Lactic Acid

Lactic acid exists in many foods, beverages, and fermented products, such as yogurt and butter (Soukoulis et al., 2007; Gamella et al., 2010; Shapiro and Silanikove, 2010). The L-lactic acid indicates the condition of eggs incubated for spoilage, intensive care, and surgical procedures (Frost and Meyerhoff, 2006; Ispas et al., 2012). An electrochemical sensor for the detection of lactic acid has been successfully developed.

**Figure 8:**
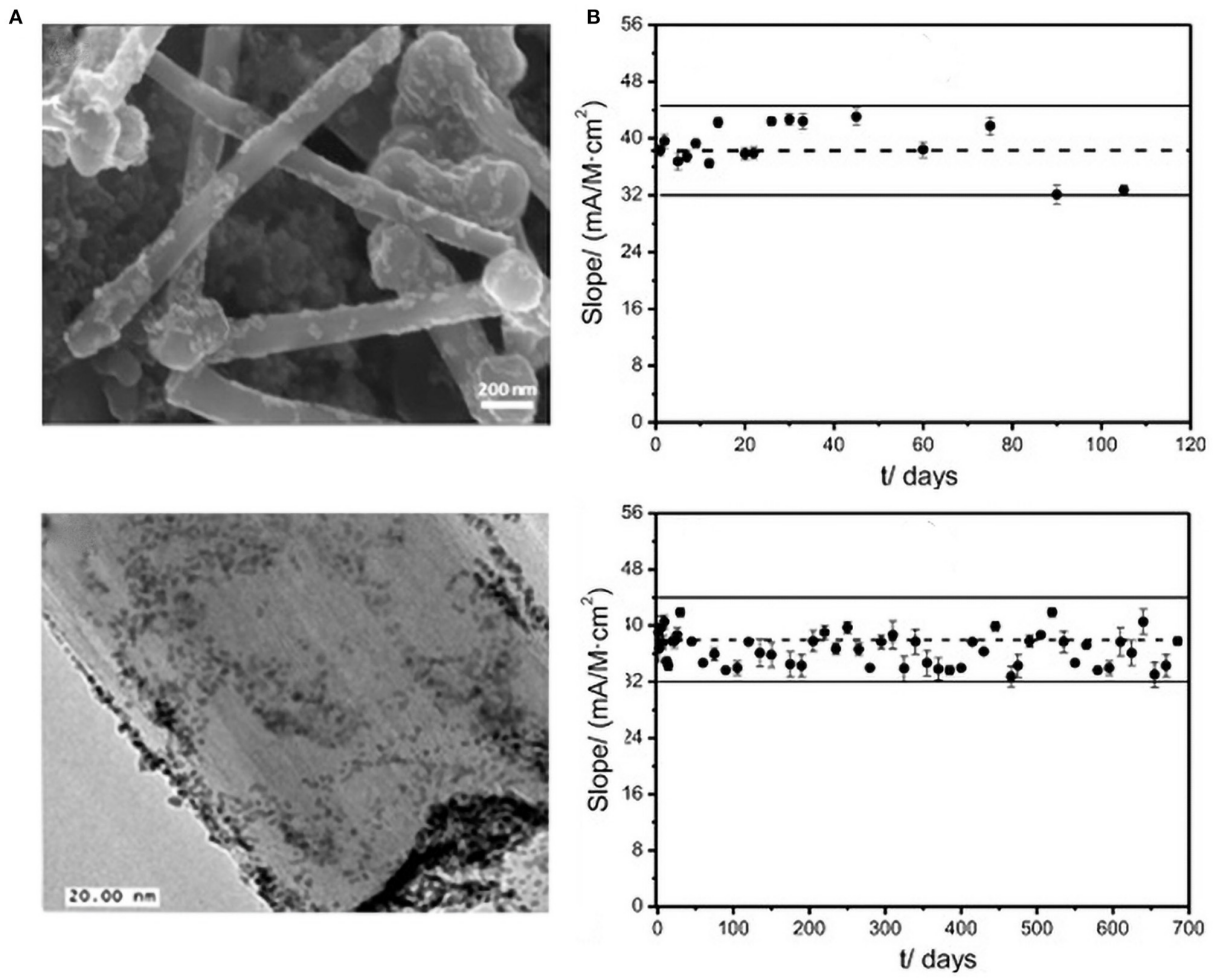
**(A)** FESEM and TEM of the PtNp/GCNF nanoparticles. **(B)** Control diagram of the PtNPs/GCNF-PEI-FA-LOx-Gly-SPCE biosensor under room temperature conditions. At −20°C, the upper limit is three times of the standard deviation of the limit slope (*n* = 10), electrolyte: 0.1 M PB, pH = 7. Eapp = +0.3 V (error bar, *n* = 5) (Chen et al., 2004). Reproduced with the permission of 2014 Elsevier B.V.

For instance, Loaiza et al. (2015) overcame the shortcomings of traditional disk electrodes (such as surface regeneration after each measurement) by developing an amperometric lactic acid biosensor based on hybrid nanomaterials and screen-printed electrodes (SPE) (Lucarelli et al., 2004; Loaiza et al., 2008). They first developed PtNPs/GCNF before chemically reducing Pt precursors on the surface of graphitized carbon nanofibers (GCNF) (Chen et al., 2004). Thereafter, they immobilized the PtNPs on the PtNPs/GCNF, and constructed biosensors by covalently immobilizing lactate oxidase (LOx) (Figure 8A). The sensor recorded good repeatability (RSD 4.9%, *n* =10). After 3 months of storage at room temperature, 90% of the signal was retained. After 18 months of storage at −20°C, the sensor performance was 95% efficient (Figure 8B).

In contrast, Liu et al. (2009) used platinum-black nanoparticles and ferricyanide modified gold film to prepare electrodes which could detect L-lactic acid in serum. The electrodes had a wide linear detection range (1–20 mM), high sensitivity (1.43 μA mM^−1^), and rapid detection time (50 s). Even after storage at room temperature for one year, its activity remained at more than 90%. In a related research, Yu et al. (2013) prepared a dioxidase [glucose oxidase (GOx), L-lactate oxidase (LOD)] biosensor by combining electrochemical materials and an *in vivo* microdialysis system. The functional Pt nanoparticles possessed adjustable mesoporous carbon particles and could simultaneously detect glucose and L-lactic acid in brain microdialysate.

At present, the detection range of glucose sensors for human and animal bodies is narrow, reaching only dozens of millimoles per liter, it also cannot be used for the detection of plant glucose. Therefore, the combination of sensors and nanomaterials must be further optimized. By introducing multiwalled carbon nanotubes (MWCNTs) to stabilize enzymes or introducing other nanomaterials or changing modification methods, the practical application of integrated sensing systems can be extended in medicine and agricultural production.

### Detection of NADH

As a cofactor involved in many enzymatic reactions, NAD+ is an essential substrate in medical, chemical, and biological processes. Efficient bioelectrocatalysis of NADH electrochemical oxidation contributed to the development of dehydrogenase amperometric sensitization systems (Radoi and Compagnone, 2009).

Jimenez et al. (2014) developed novel amperometric biosensors by electrodepositing polymer membranes on the Pt or PtNPs/Pt electrodes, and then immobilized alcohol dehydrogenase (ADH) on the electrodes by covalent cross-linking. The polymer/PtNPs/Pt electrode can withstand a linear concentration range of 25 mM (r = 0.9979) and 21 mM (r = 0.99849). The electrode has a detection limit of 4.78 and 6.18 μM for PDAMS/PtNPs/Pt and PMDUS/PtNPs/Pt, respectively, and a sensitivity of 68.24 and 40.21 μAmM^−1^cm^−2^. It has a linear range of 30 mm, and a sensitivity of 0.957 or 0.756 μA mM^−*l*^ cm^−2^ toward ADH/PDAMS/PtNPs/Pt or ADH/PMDUS/PINPSP, respectively.

In the oxidation process of NADH directly detected by the bare electrode, the electron transfer rate is very slow and highly irreversible. It requires a high activation energy and produces a very high oxidation overpotential. In addition, it is more difficult to detect NADH in real-time biological samples owing to the more complex composition of biological samples. Using the electrochemical method to detect NADH at a low oxidation potential has therefore become an urgent problem to be solved.

### Detection of Xanthine

Gout, xanthinuria, and hyperuricemia are all associated with purine metabolism (Joshi et al., 2018). Electrochemical analysis of xanthine is based on the oxidation of H_2_O_2_ or dielectric redox reactions (Shan et al., 2009).

**Figure 9:**
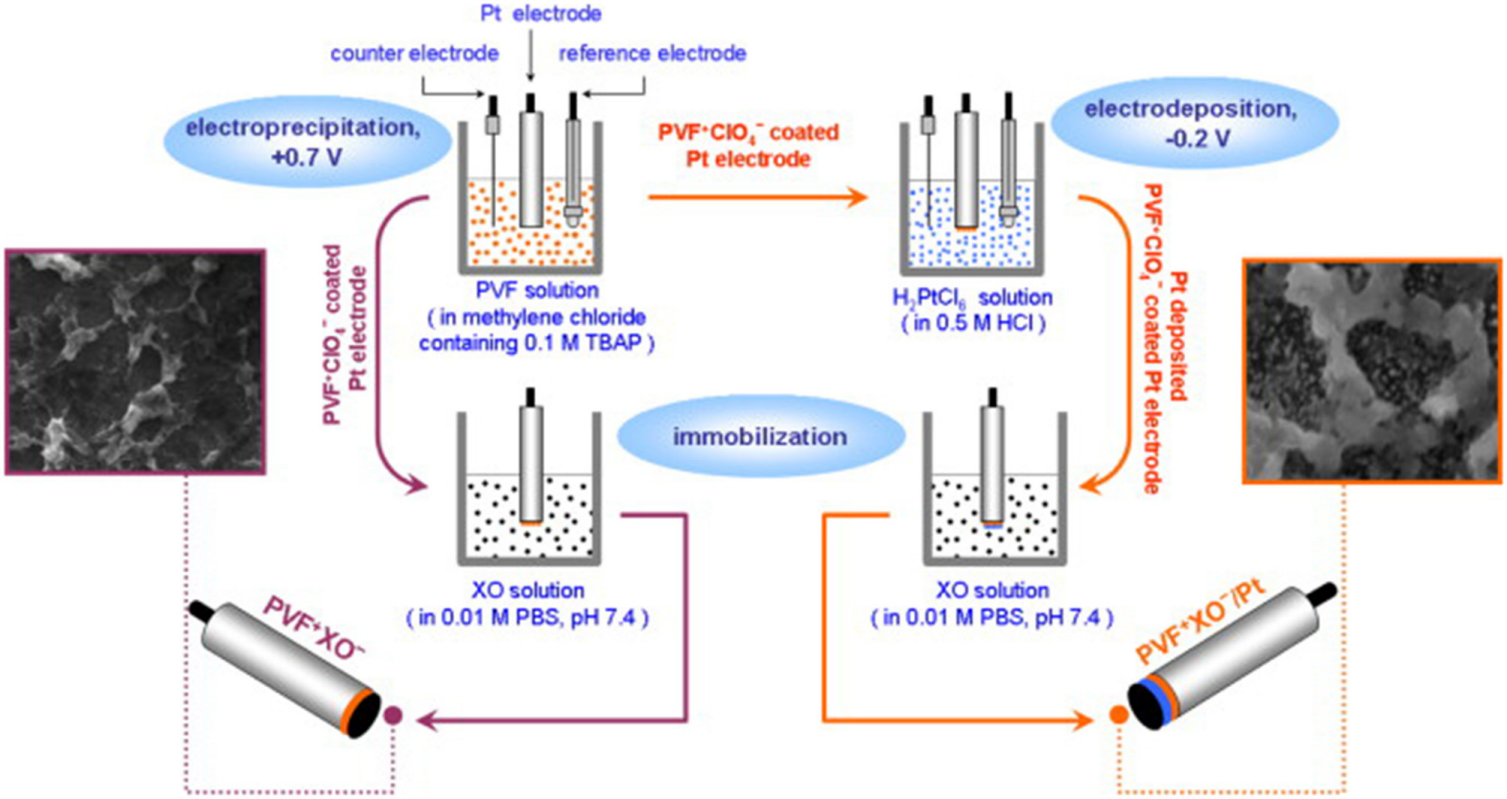
Schematics showing the development of PV FXO and PV FXO-/PT electrodes (Bas et al., 2011). Reproduced with the permission of 2011 Elsevier B.V.

Bas et al. (2011) made a xanthine biosensor by immobilizing xanthine adenylate on a polyvinyl alcohol ferric ceria perchlorate matrix (PVF^+^ClO_4−_) (Figure 9). The linear range of the PV FXO^−^/PT is 0.43 × 10^−3^–2.84 mM, PV FXO^−^, detection limit of 1.73 × 10^−3^–1.74 mm, and PV FXO^−^/PT is 0.43 × 10^−3^–2.84 mM. The current response of PVF^+^XO^−^/Pt is 42% of the initial value at 25 days, thereby providing excellent stability.

In addition, xanthine, biomolecules including dopamine, uric acid, hypoxanthine, and ascorbic acid that have a high and close oxidation overpotential on conventional glassy carbon electrodes, are easily absorbed along with their oxidation products on the surface of the electrode, and cause pollution on the electrode surface, which greatly affects the analytical effect of the detection.

### Detection of Ascorbic Acid

As a cofactor of numerous enzymes, ascorbic acid (AA) is an important biological compound in human metabolism. To date, several electrochemical, spectrophotometric, chromatographic, and colorimetric methods for AA detection have been developed (Malashikhina and Pavlov, 2012; Singh et al., 2012).

Dursun and Gelmez (2010) modified a glassy carbon electrode by lining nano-platinum on multiwalled carbon by electrochemical deposition (Pt nanoparticles modified MWCNT/GCE). The detection limits of the sensor against ascorbic acid (AA), dopamine (DA), and uric acid (UA) were 1.9 × 10^−5^, 2.78 × 10^−8^, and 3.2 × 10^−8^ M, respectively. The lowest detection limits of the sensors against AA, DA, and UA were 2 × 10^−5^, 4.83 × 10^−8^, and 3.5 × 10^−7^ M, respectively.

In recent years, colorimetry has attracted greater attention due to its simplicity, speed, and low cost. Wang et al. (2017) prepared CuO nanosheets through hydrothermal synthesis, and nanoplatinum through the NaBH_4_ method. Using CuO nanosheets as reducing agents, they designed a uniform and stable CuO–Pt nanocomposite, which relied on colorimetry to detect AA.

Compared with the instability of the enzyme electrode, the enzyme-free electrode is much less restricted; however, ascorbic acid generally coexists with dopamine and uric acid, and the interference of both against ascorbic acid is very great, which makes the sensitivity and selectivity of ascorbic acid detection very low. The development of an ascorbic acid sensor capable of efficient and independent detection is therefore still an important research direction.

### Detection of Urea

Excessive accumulation of urea in serum can cause shock, dehydration, and kidney diseases. As such, accurate detection of urea is critical in the clinical management of numerous complications. Biosensors offer a rapid alternative for urea detection, given their high sensitivity, specificity, and long-term performance.

Hosseinian et al. (2019a) used Ni(NO_3_)_2_ and sodium hydroxide (NaOH) solution to coat NiO NP, NiO NP, and polypyrrole (PPy) on a platinum electrode (Pt/PPy–NiO electrode) for detecting urea. The electrode had a high sensitivity of 0.153 mAmM^−1^cm^−2^), a good linear response of 0.7–26.7 mM, *R*^2^ = 0.993), and satisfactory selectivity.

Macroporous polypyrrole (MPPy) and pyrrole were also used to prepare a urease - coated platinum electrode (Hosseinian et al., 2019b). The linear response of the polypyrrole (PPy) electrode ranged between 1.67 and 8.32 mM (*R*^2^ = 0.99), whereas the sensitivity was 0.0035 mA mM^−1^. The detection limit was 2.57 mM whereas the response time was 7 s. The XYZ of the MPPy electrode were 0.5–10.82 mM (*R*^2^ = 0.99, 0.0432 mA mM^−1^ and ~5 s, respectively.

As it is difficult to exploit the various characteristics of platinum nanoparticles due to complexity of the electrode process and the limitation of detection time, the research and development of a platinum-based urea biosensor should follow certain steps. First, the development should adopt simple methods to improve anti-interference performance. Second, it should further explore the testing of blood samples. Third, it should use compound nanoparticles to enhance the activity of urease. Last, it should adopt simple and effective methods to improve the stability of the sensor and meet commercial needs.

### Detection of DNA

DNA biosensors are widely used in drug analysis, environmental monitoring, and clinical diagnosis of diseases because of their low cost, rapidness, and specificity (Yin et al., 2012). Platinum nanoparticles have been used in the preparation of DNA sensors because of their high catalytic and adsorption capacity.

Wang et al. (2008) prepared a DNA biosensor by lining platinum nanoparticles (Pt-Nano) on the surface of a glassy carbon electrode (GCE) through electrodeposition. The linear calibration concentration of the complementary DNA ranged between 14 × 10^−9^–2.14 × 10^−7^ M, whereas the detection limit and relative standard deviation were 1 × 10^−9^ M and 5.89% (*n* = 5), respectively. Although the performance of the sensor is highly reproducible, its sensitivity has not been verified yet. To improve sensitivity and detection limit. Yin et al. (2012) used Langmuir–Blodgett and thermal reduction of silicon (Si/SiO_2_) to prepare a thin film of graphene oxide (rGO). Through the photochemical reduction of PtNPs, they recovered graphene oxide (rGO) and PtNPs/rGO composite materials. The composite sensor was highly sensitive (2.4 nM) and rapid in the detection of single-stranded DNA.

**Figure 10:**
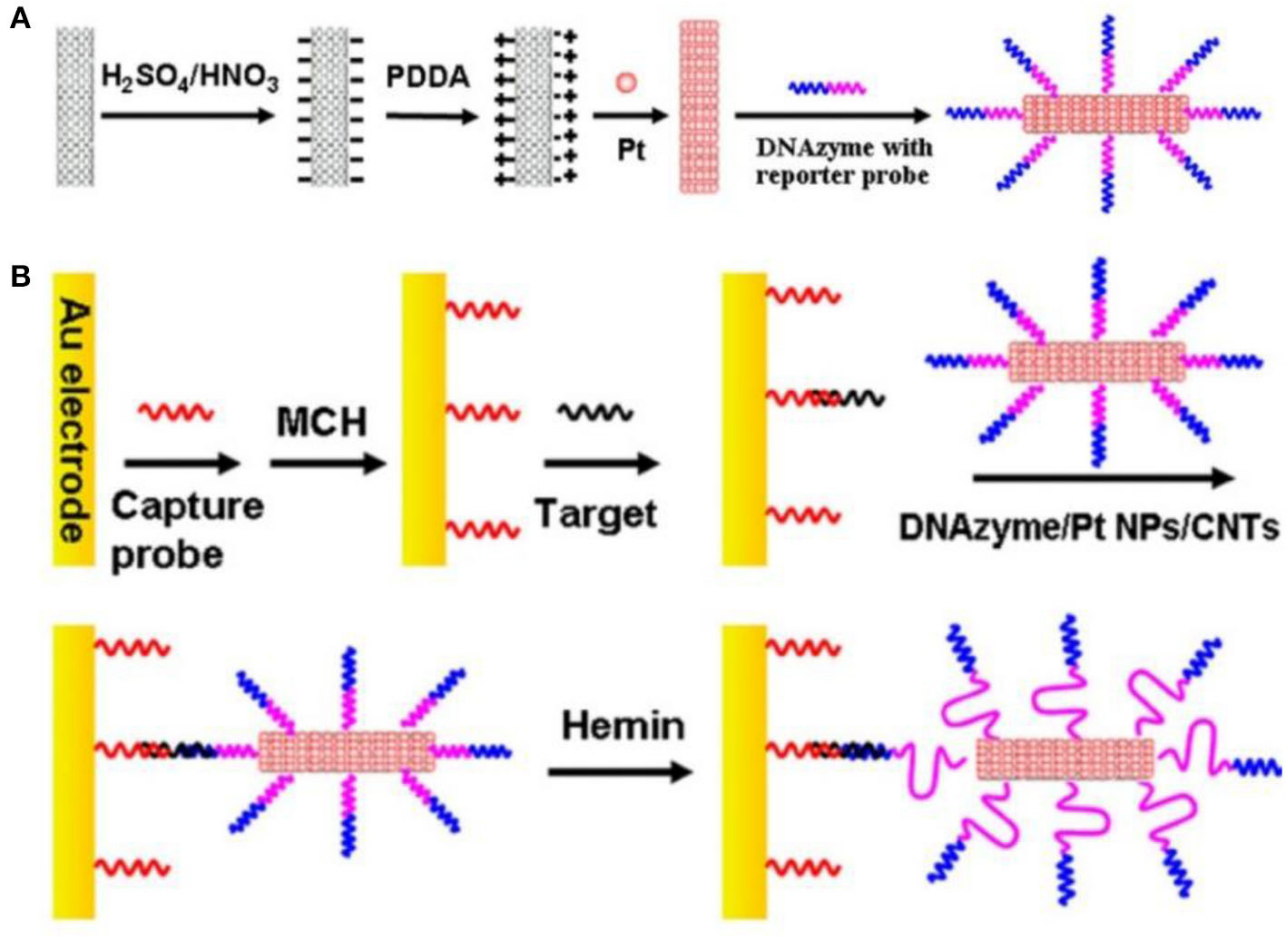
**(A)** Schematic flow for the preparation of DNAzyme/PtNPS/CNTs bioconjugate. **(B)** Preparation and sandwich detection module of DNA sensor (Dong et al., 2012). Reproduced with the permission of 2012 Elsevier B.V.

Dong et al. (2012) prepared PtNPs using the Polsky's method (Polsky et al., 2006). Here, PtNPs were coated with a polyelectrolytes layer of carboxylated CNTs (Figure 10A), and thereafter attached DNA enzymes and DNA probes through platinum-sulfur bonds (Figure 10B). The trace DNA detection sensor displayed the linear detection range of 1.0 fM−10 pM and the detection limit of 0.6 fM. The sensor was relatively specific, reproducible, and stable.

Daneshpour et al. (2016) first synthesized Fe_3_O_4_ magnetic nanomaterials using Mahdavi's method (Mahdavi et al., 2013) and thereafter prepared Fe_3_O_4_/TMC composite nanomaterials by coating the Fe_3_O_4_ with trimethyl chitosan (TMC). They then prepared colloidal gold nanoparticles using the Gole method (Gole and Murphy, 2004), particularly by reducing HAuCl_4_. Free Au was then reacted with Fe_3_O_4_/TMC, generating the Fe3O4/TMC/Au composite nanomaterials for DNA probing. Biosensor for methylated DNA has been prepared using polythiophene (PT). The concentration detection range of this method is 1 × 10^−14^–5 × 10^−9^ M, whereas the detection limit is 2 × 10^−15^ M.

**Figure 11:**
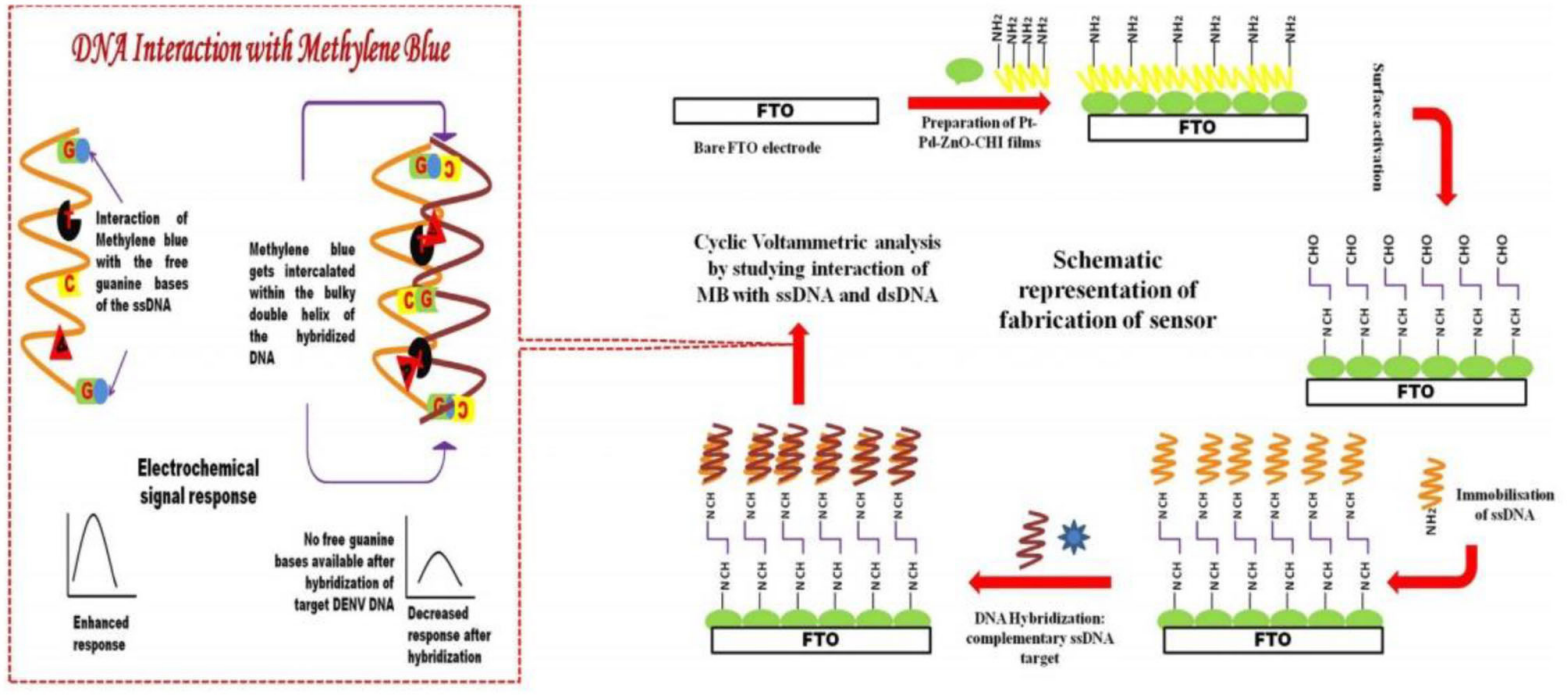
Schematic flow for the preparation and detection principle of DNA sensors (Singhal et al., 2017). Reproduced with the permission of 2017 Elsevier B.V.

Infection due to the dengue virus can cause highly fatal dengue hemorrhagic fever and dengue shock syndrome (Nguyen et al., 2013; Ramanan et al., 2021). Singhal et al. (2017) used the one-pot method to synthesize Pt–Pd nanoflowers, and thereafter added sodium hydroxide to zinc nitrate to prepare nano-zinc oxide (ZnO NPs). The nano-zinc oxide was then mixed with Pt–Pd to generate ZnO/Pt–Pd nanocomposites. The nanocomposites were lined on the FTO electrode before coating with the DNA (PDNA) probe. The sensor could detect the dengue virus DNA stained with methylene blue (MB) (Figure 11). The detection linear range of the sensor is 1 × 10^−6^–100 × 10^−6^ M, whereas the the limit of detection(LOD) and limit of quantification (LOQ) 4.3 × 10^−5^ and 9.5 × 10^−5^ M, respectively.

The working principle of platinum-based DNA biosensor is based on nucleic acid hybridization reaction. However, hybridization time and hybridization amount are a pair of contradictory factors. Although the sensitivity of platinum-based DNA biosensor is within the acceptable range, the response time is too long.

### Immune Marker

Coronary heart, cerebrovascular, rheumatic heart, and congenital heart diseases are among the most common cardiovascular diseases globally. Serum markers are critical in diabolizing cardiac diseases (Purins et al., 2010; Qureshi et al., 2012). Among them, myoglobin is a potentially reliable early diagnostic marker for the diagnosis of acute myocardial infarction (AMI) (Statland, 1996). Mishra et al. (2014) coated carbon diimide on coupling reaction of the three-dimensional carboxyl functional (MPA) Pt nanoparticles with myoglobin antibody immobilized on indium tin oxide coated glass, to develop Ab-cMb/Pt (MPA)/APTES (a 3-aminopropyltriethoxy silane)/ITO (indium-tin-oxide) glass bioelectrodes. The linear detection range and sensitivity of the cardiac myoglobin immune sensor are 0.01–1 μg ml^−1^ and 184.8 Ω cm^2^, respectively.

Carcinoembryonic antigen is a glycoprotein produced by cancer tissues. The antigen is mainly found in the digestive system, and causes an immune response in many patients. The antigen is a good biomarker for monitoring the prognosis and therapy response of colorectal, breast, and lung cancers (Castano-Guerrero et al., 2021; Ma et al., 2021). Fu et al. (2017) first prepared an Au/PtNPs biological probe, then mAb1-MBs using mAb1 and MBs, and finally, a barometer-based immunosensor to detect the carcinoembryonic antigen (CEA) and ractopamine (RAC). Thrombin and mercury ion (Hg^2+^) sensors based on this principle have also been developed. The linear detection ranges of CEA, RAC, thrombin, and Hg^2+^ biosensors were 0.025–1.6,0.0625–4, 4–128, and 0.25–16 ng/ml, respectively. Their respective detection limits were 0.021,0.051, 2.4, and 0.22 ng/ml, respectively.

**Figure 12:**
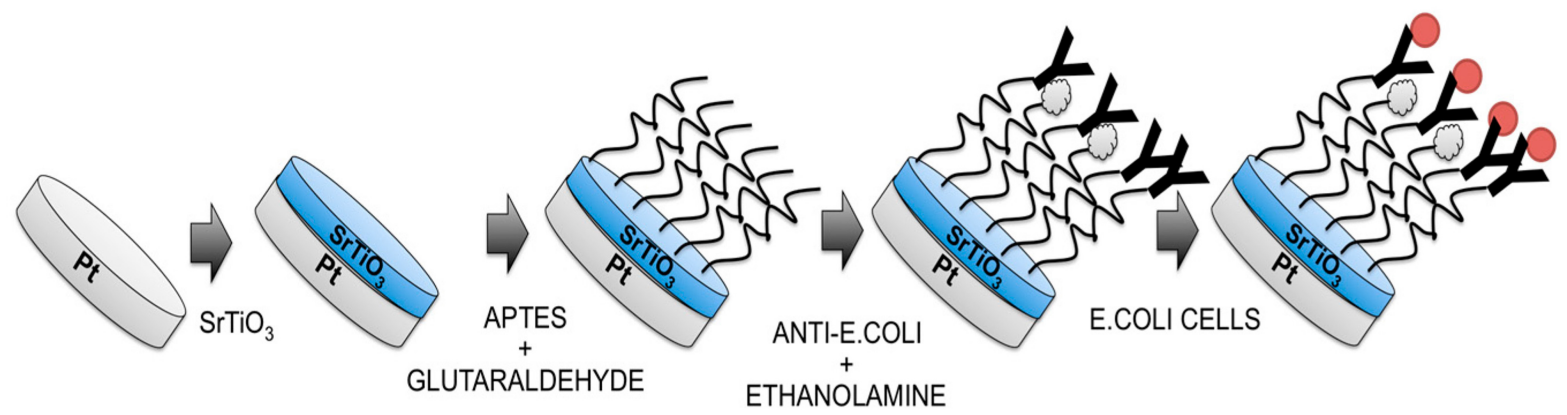
Diagrammatic flow for the preparation of *Escherichia coli* O157:H7 immunosensor (Malvano et al., 2018). Reproduced with permission from the Multidisciplinary Digital Publishing Institute.

For the first time, Malvano et al. (2018) used strontium perovskite layer (SrTiO_3_) as biosensors against immune biomarkers. Here, SrTiO_3_ was coated on the platinum electrode and used in the detection of *Escherichia coli* O157:H7 (Figure 12). The detection limit of the sensor is 1–7 log CFU/ml.

In a related research, Zhu et al. (2018a) prepared Escherichia coli O157:H7 biosensor using old-platinum core/shell nanoparticles (Au/PtNPs) with different Pt shell thicknesses by changing the molar ratio of H_2_PtCl_6_ to HAuCl_4_ in solution. The *E. coli* O157:H7 was detected using Silica-coated magnetite nanoparticles (Fe_3_O_4_) sandwich immunosensor. The detection range of the biosensor was 4 × 10^−2^–4 × 10^−8^ CFU·ml^−1^, whereas the linear detection limit was 91 CFU·ml^−1^.

Mumps is a common childhood infectious disease caused by the mumps virus. In serious scenarios, mumps cause orchitis and permanent deafness (Galazka et al., 1999). Long et al. (2020) synthesized Au NRs using the seed-mediated method. The compound was then added in a mixture of K_2_PtCl_4_ and ascorbic acid, generating Au/PtNRs. Homogeneous Au/Pt nanorods and mesoporous SiO_2_ nanostructures with oxidase activity were also prepared separately. The mumps virus could be diagnosed using enzyme-linked immunosorptive assay (ELISA), at the detection limit of as low as 10 ng/ml.

The strong adsorption and catalytic performance of Pt nanoparticles equip the electrochemical immunosensor with a high affinity for antigen and antibody reactions, and a high sensitivity to the receptor, which can realize the detection of low-concentration samples and multiple markers. However, at present, immunosensors prepared by using platinum-based functionalized composites can only be fixed to detect one or several simple disease markers. If the body produces a cross-immune response, the accuracy and sensitivity of the detection results are greatly reduced, which is another problem that needs to be solved urgently in future research on platinum-based immunosensors.

## The Applications of the PtNP-Based Biosensors in Food Fields

The food industry is the key application field of nano-biosensors. Functional platinum nanomaterials can be used as catalysts, immobilization platforms, and optical fiber labels. Improving the performance of the biosensor can afford it higher sensitivity, stability, and selectivity. Platinum nanomaterials can modify not only biomolecules to provide specific targeting, but also enhance the immobilization of biomolecules on the electrode surface. These characteristics can stimulate joint research on biosensors, platinum nanomaterials, and biosensors based on platinum nanomaterials, which are widely used in food detection.

### Detection of Ethanol

Ethanol is an organic compound that can be used in the manufacture of acetic acid, beverage, essence, dye, and fuel, among others. Ethanol is also applied in the defense, food, medical, and health industries; synthesis of organic compounds; and agricultural production (Gesheva et al., 2012; Jagadale et al., 2014).

To prepare biosensors for ethanol in alcoholic beverages, Ozdokur et al. (2016) prepared GCE/MnOx–MoOx electrode through potential deposition technology by soaking freshly polished electrode in MnSO_4_, Na_2_MoO_4_, and Na_2_SO_4._ The compound was then immersed in ${\text{PtCl}}_{4}^{2-}$ solution to generate GCE/MnOx–MoOx/Pt, an effective ethanol biosensor. The detection range of the biosensor was 0.075–5 mM, with a response time of 63 s.

### Detection of Natamycin

Natamycin is a polyene amphoteric macrolide which prevents or delays the growth of yeast and mold on surfaces. As such, it is widely used in treating fungal infections and preservation of meat products, cheese, wine, and other beverages (Cong et al., 2007; Alberts et al., 2011). This underscores the need to accurately determine natamycin concentration in food samples. Several analytical techniques such as spectrophotometry, high-performance liquid chromatography, and liquid chromatography-mass spectrometry have been used in determining natamycin concentration (Koontz et al., 2003; Repizo et al., 2012). However, these analytical techniques are expensive and slow. Contrarily, electrochemical methods are simple, highly sensitive, specific, and cheap (Yousefi et al., 2018); however, only few studies have explored the utility of electrochemical methods in determining natamycin concentration.

Yousefi et al. (2018) first prepared platinum-coated CdS nanoparticles using the microwave-assisted method. Composite materials were then prepared using MWCNTs and Pt-coated CdS. The carbon electrode (SPCE) was then mixed with MWCNTs and the Pt-CdS to form MWCNTs-Pt-doped CdS nanoparticles/SPCE. The detection range of the sensor is 0.2–70 μM, whereas its detection limit is 0.12 μM. It is also stable, sensitive, and consistent. The sensor has been effectively used in determining natamycin concentration in commercial yogurt, beverages, and cheese samples.

### Detection of Salmonella

Salmonella is one of the most common foodborne pathogens (Chen et al., 2019). Traditional detection methods are slow and complex. Although enzyme immunoassay and real-time polymerase chain reaction (PCR) are highly sensitive and stable, they are relatively complex (Lee et al., 2015; Ma et al., 2018).

**Figure 13:**
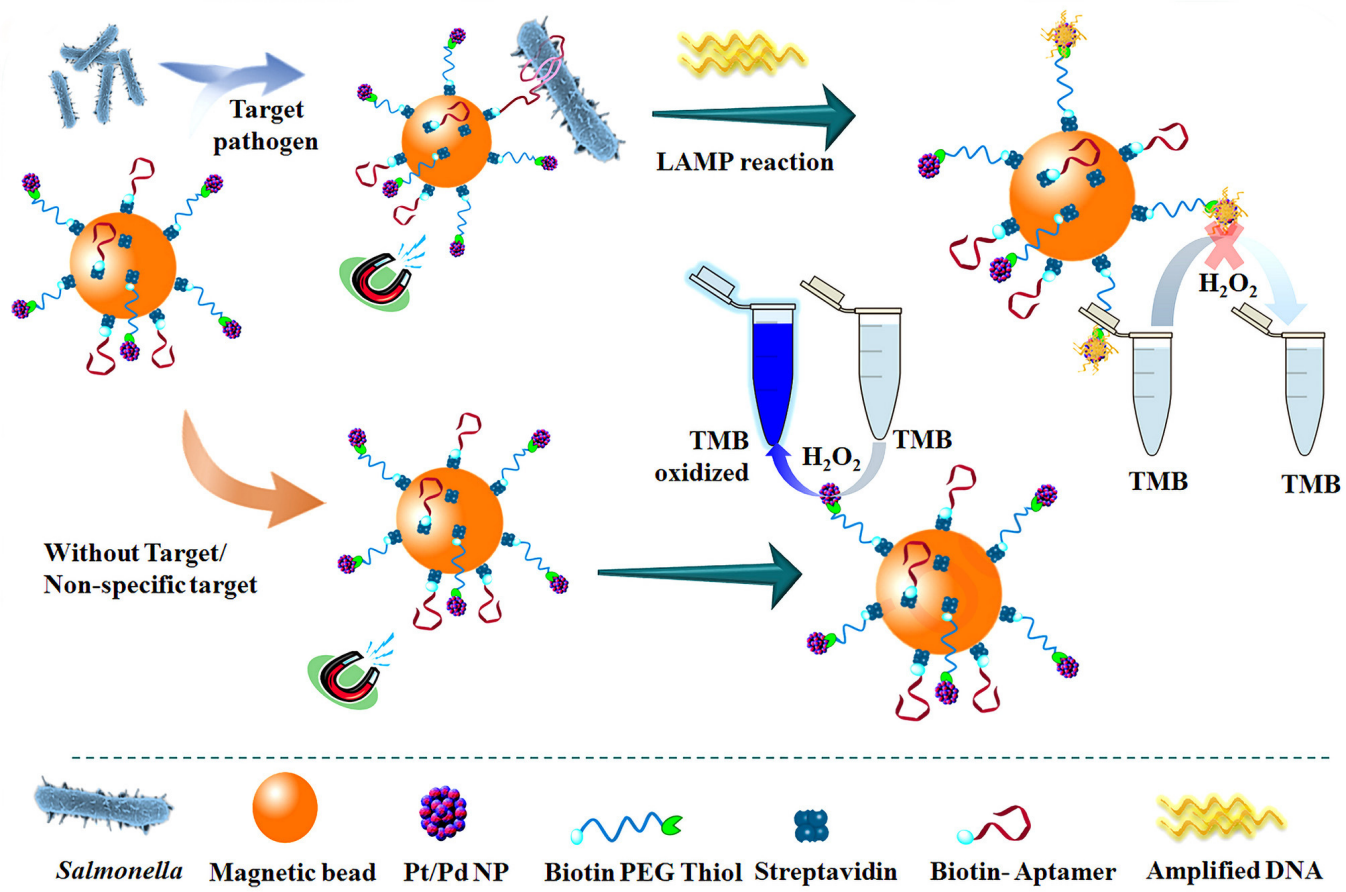
The schematic flow for the detection of *Streptococcus typhimurium* using DNA-mediated inhibition of Pt/PdNP simulated peroxidase activity. The inhibition is induced by the bifunctional nanobiological probe catalysts in combination with LAMP reaction (Dehghani et al., 2021). Reproduced with the permission of 2020 Elsevier Ltd.

Dehghani et al. (2021) used platinum-coated magnetic beads/palladium nanoparticles (Pt/PdNP) and conjugated them with DNA aptamers for biological probing (Figure 13). It was combined with loop-mediated isothermal amplification (LAMP) for the detection of salmonella in food and fecal samples. The test can accurately detect salmonella typhimurium in chicken samples within less than 3 h, even at concentrations as low as 10–15 and 3–10 CFU/ml in egg and chicken fecal samples.

### Detection of *Escherichia coli*

*Escherichia coli* is a common foodborne pathogen, responsible for numerous food and waterborne disease outbreaks (Hassan et al., 2015). Strain O157:H7 is one of the most virulent strains of *Escherichia coli* bacteria, which can cause hemorrhagic colitis and hemolytic uremic syndrome (Lin et al., 2010). Currently, *E. coli* 0157:H7 is detected using enzyme-linked immunosorbent assay (ELISA) and polymerase chain reaction (PCR), among other techniques, which are slow and very expensive (Kurins et al., 2010; Roda et al., 2012; Gan et al., 2017; Ye et al., 2017). Electrochemical biosensors, immunochromatography, and immunomagnetic separation are processes that are simple, quick, and highly sensitive.

Zhu et al. (2018b) first coated Fe_3_O_4_ magnetic nanoparticles with silica (Fe_3_O_4_@SiO_2_), and thereafter coated neutral red (NR) with Au/Pt nanoparticles using functionalized graphene to form rGO–NR–Au/Pt composites. The two compounds were then mixed to form a non-enzyme sandwich electrochemical immunosensor. The concentration of detection range was 4 × 10^3^–4 × 10^8^ CFU ml^−1^, whereas the limit of detection was 4.5 × 102 CFU ml^−1^. The method is effective in identifying *E. coli* in pork and milk samples.

Currently, there are still many types of biosensors emerging based on the Pt nanomaterials. In addition to the Pt nanomaterials, there are sensors based on Pt oxide and Pt alloy which would can be used in real life in the future (Du et al., 2002; Wu et al., 2016; Li et al., 2017).

Some problems remain to be solved in biosensors based on platinum nanomaterials in the food industry. First, the test sample status is limited. Most platinum-based biological sensors only apply to the detection of liquid samples, such as milk, drinks, and yogurt. There are only a few applications in the detection of meat, and applying them to the base of complex food may cause serious interference. Future research should therefore pursue platinum-nano material that has excellent performance on the basis of the development of biological sensors to reduce sample pretreatment. Second, there is a single test sample type. Most platinum-based biosensors can only detect a single pathogen, so biosensors that can meet the requirements of multiple microbial detections are often needed in food quality control. We should therefore choose more specific receptors and a more sensitive substrate material.

## Summary and Outlook

In this paper, we have reviewed the applications of Pt-based electrochemical biosensors with various applications in agriculture, industry, and medical fields. Of note, Pt nanoparticles, due to their high specific surface area, excellent electrical conductivity, and decent biocompatibility, are good candidates for the assembly of biosensors. Many different sensors were thereby orchestrated based on the advantages of PtNPs; however, toward the requirements in the practical applications, more strategies should be explored to enhance sensitivity, selectivity, and versatility.

In terms of sensitivity, currently, electrochemical techniques are good choices; however, sensitivity would be severely influenced by the detection conditions. We should therefore choose more specific receptors and more sensitive substrate materials such as complete bacterial cells, quantum dots, graphene, multi-walled carbon nanotubes, magnetic beads, and adaptation. The resulting choice of receptor and substrate material should then be combined with the current advanced nanotechnology (pulse potential deposition technology), 3D technology, CRISPR technology, microfluidic technology, nanometer particle collision electrochemical, and intelligent packaging that can successfully build sensitive biological analysis tool. There is also a need to develop a deeper theoretical research, for the preparation of a better performance of electrode, and a better understanding of emerging materials and mechanism of the interaction of biological molecules.

In terms of selectivity, the most prevalent target principle currently being used is the covalent bonding between the target and PtNPs. The bonding is not specific; thus, leading to misdetection or false positive results. To improve this part, more selective bonding molecules should be adopted, such as antigen and antibody, biotin and avidin, or adamantane and cyclodextrin.

In terms of convenience and portability, in the future, platinum-based nanomaterial biosensors should be easy to carry and standardized to improve the utilization rate. Portability can be enhanced by connecting and integrating electrochemical sensors with electronic products used in everyday life, such as mobile phones, tablets, and computers.

As to versatility, given the ancillary cost of PtNPs, some composites based on PtNPs should be explored to further lower the cost and enhance other properties such as polymer nanomaterials (polymer-PtNPs/Pt), carbon nanotubes nanomaterials (Pt-CNT), and graphene/graphene oxide nanomaterials (GO-Pt).

## Author Contributions

HY drafted the manuscript. XN, JY, LL, YZ, and SX guided and amended the manuscript. YS and KS helped to review the manuscript. All authors contributed to the manuscript.

## Conflict of Interest

The authors declare that the research was conducted in the absence of any commercial or financial relationships that could be construed as a potential conflict of interest.
